# Effects of first radioiodine ablation on functions of salivary glands in patients with differentiated thyroid cancer

**DOI:** 10.1097/MD.0000000000007164

**Published:** 2017-06-23

**Authors:** Arun Upadhyaya, Zhaowei Meng, Peng Wang, Guizhi Zhang, Qiang Jia, Jian Tan, Xue Li, Tianpeng Hu, Na Liu, Pingping Zhou, Sen Wang, Xiaoxia Liu, Huiying Wang, Chunmei Zhang, Fengxiao Zhao, Ziyu Yan

**Affiliations:** Department of Nuclear Medicine, Tianjin Medical University General Hospital, Tianjin, P.R. China.

**Keywords:** differentiated thyroid cancer, radioactive iodine (^131^I) therapy, salivary gland

## Abstract

The aim of this study was to evaluate the effects of the first radioactive iodine (^131^I) therapy on functions of salivary glands in patients with differentiated thyroid carcinoma (DTC).

There were 36 consented patients with DTC enrolled in this study, who received 3.7 GBq (100mCi) ^131^I for ablation after total thyroidectomy. Salivary gland function was assessed using salivary gland scintigraphy in two phases, one 4 hours before and the other 6 months after ^131^I therapy (both under thyrotropin stimulation condition). Quantitative parameters including uptake fraction (UF), uptake index (UI), excretion fraction (EF), and excretion ratio (ER) were measured and compared. Blood parameters were also compared. Associations between sex and outcome of the first ^131^I therapy as well as individual salivary gland function were measured. Wilcoxon Signed Rank Sum test and *χ*^2^ test were used for statistical analysis.

When compared between pre-ablation and post-ablation, UF of bilateral parotid and submandibular glands were significantly increased (all *P* < .01). UI of both submandibular glands were significantly increased (*P* < .05). This seemingly increased uptake function after the first ^131^I therapy was actually compensatory mechanism of salivary gland, which indicated a possible intermediate state after radiation. But salivary glands’ secretory function had not changed significantly except for left submandibular gland; we demonstrated that only left submandibular gland showed significantly decreased ER (*P* < .05). Thyroglobulin and thyroglobulin antibody significantly decreased after ^131^I therapy (*P* < .05). There were no sex differences on therapeutic outcome and salivary gland dysfunctions after the first ^131^I therapy. Salivary gland of both males and females could be affected by ^131^I therapy.

The first ^131^I ablative therapy may impair the salivary uptake and secretory function of patients with DTC. There was no association between sex and salivary gland dysfunction.

## 1 Introduction

Differentiated thyroid cancer (DTC) is one of the most common endocrine malignancies, which is currently estimated to be the fifth most common cancer in women in the United States.^[1]^ The use of radioactive iodine (^131^I) for the ablation of residual thyroid tissue after thyroidectomy is well recognized as a part of the management of DTC.^[1–3]^ Sodium iodide symporter (NIS), possessed by thyroid cancer tissues, plays a profound function in uptake of ^131^I by these tissues.^[4,5]^ Salivary gland, stomach, and breast are also noted to possess NIS and thus can take up ^131^I. It is demonstrated that the accumulation of ^131^I in salivary gland could be about 30 to 40 times to that in plasma.^[5,6]^ It is this ability to concentrate that causes glandular damage when ^131^I is used.^[7]^

Previous studies have shown that after ^131^I therapy, the salivary glands would be affected to different degrees of damage, but the reports were inconsistent. For instance, Klein Hesselink et al^[8]^ showed the salivary gland damage varied with the sensitivity of the patients to the radiation and the cumulative dose of ^131^I. Some reports demonstrated that acute sialadenitis could happen in as many as 15% of patients.^[9,10]^ In the study by Kang et al,^[11]^ only the parotid gland excretory function was reduced in post-^131^I therapy patients. Raza et al^[12]^ and Malpani et al^[13]^ reported that some patients showed no visualization of the right parotid (RP) gland, whereas the left parotid (LP) gland had some degree of parenchymal function after radioiodine treatment, and asymmetry in salivary gland function after ^131^I may have been because of an asymmetrical effective ^131^I concentration respectively. In another recent article, it was found that damage occurs only after activities higher than 5.55 GBq.^[14]^ Dosage of up to 150 mCi was not revealed to affect either uptake or secretion functions, and was likely to be a safe and potentially effective dosage that can be applied without damage to the salivary glands.^[15–17]^

To provide more evidence to clarify the above inconsistency, the present study aimed to assess and compare the salivary uptake and secretory functions of RP, LP, right submandibular (RS), and left submandibular (LS) salivary glands before and 6 months after the first ^131^I therapy in DTC patients.

## 2 Materials and methods

### 2.1 Patients’ recruitment

All patients received total thyroidectomy by our specialized thyroid surgeons with a definite pathological diagnosis of DTC. After surgery, they came to our department for ^131^I treatment. In-hospitalized DTC patients were recruited from March 2014 to April 2015, if they consented entering the study. Exclusion criteria included: patients previously treated for any other malignancies by chemotherapy and/or radiotherapy, other head and neck cancers, patients with xerostomia owing to any other reasons, other systemic disorders, patients with any deleterious habits, and patients on medications like anti-cholinergic, anti-histaminics, and other drugs causing xerostomia.

All DTC patients received ^131^I for the first time about 6 weeks after surgery. Patients were advised to take low-iodine diet for at least 3 weeks before receiving ^131^I. Before ^131^I treatment, serum parameter measurements included free triiodothyronine (FT3, reference 3.50–6.50 pmol/L), free thyroxine (FT4, reference 11.50–23.50 pmol/L), and thyroid-stimulating hormone (TSH, reference 0.30–5.00 μIU/mL, maximum 150.00 μIU/mL), which were assayed on a fully automated ADVIA Centaur analyzer (Siemens Healthcare Diagnostics, Tarrytown, NY) by chemiluminescent reaction principle. Thyroglobulin (Tg, reference 0–55.00 ng/mL) and thyroglobulin antibody (TgAb, reference 0–40.00 IU/mL) were also assessed by on a fully automated IMMULITE 2000 analyzer (Siemens Healthcare Diagnostics, Los Angeles, CA). All included patients received 100 mCi of ^131^I. All participants were then followed for at least 6 months, when another thorough assessment was conducted. Management procedures for DTC patients were reported by our group previously.^[18–23]^

The institutional review board of Tianjin Medical University General Hospital approved the ethical, methodological, and protocol aspects of this investigation. All participants provided their written informed consents. We confirm that all methods were carried out in accordance with the relevant guidelines and regulations.

### 2.2 Salivary gland imaging protocol

Preablation salivary imaging was performed in the morning under TSH stimulating condition, 4 hours prior to the first ^131^I intake. Patients were asked to fast before salivary imaging. They were positioned supinely with the neck hyperextended and imaged by using a SPECT/CT machine (Discovery NM/CT 670, General Electric Medical Systems) with a low-energy parallel hole high-resolution collimator, peak 140 kev and the window width of 20%. Each patient received a bolus intravenous injection of 370 MBq ^99m^Tc-pertechnetate via cubital vein. Immediately after administration, sequential dynamic images were taken at minute/frame on a 256  × 256 matrix for 30 minutes with zoom 1.5. At the 20^th^ minute after injection, vitamin C 0.2 g was given to chew quickly and squish it orally and sublingually for about 1 minute. Before and after injection, the radioactive counts in the syringe were measured to calculate the exact radioactivity in the body.

Patients were subjected to scintigraphy again as per the aforementioned procedure 6 months after the first ^131^I therapy, when another thorough assessment was conducted. This salivary imaging was also performed under TSH stimulating condition, either during a diagnostic ^131^I scan or another ^131^I therapy 6 months after the first ^131^I therapy.

### 2.3 Image analysis

Circular regions of interest (ROIs) were drawn manually over each of the parotid and submandibular glands. A similar uniform background region was drawn in bilateral temporo-orbital regions for each parotid glands and the bilateral supraclavicular regions for each submandibular glands. Time activity curves of uptake and washout of ^99m^Tc-pertechnetate were generated using counts per minute. On the basis of these ROI counts and subsequent time activity curve, the following functional indices were derived for each salivary gland by the following modified formulas^[24–26]^:

(1) maximum uptake fraction (UF)
  UF  =  (count of a salivary gland at maximum minute – count of the background of corresponding salivary gland at maximum minute) / (count of syringe per minute before use – count of syringe per minute after use × 100%;
(2) uptake index (UI)
  UI  =  (count of a salivary gland at the maximum uptake minute – count of the background of corresponding salivary gland at the maximum uptake minute) / count of background of the salivary gland at the maximum uptake minute;
(3) excretion fraction (EF)
  EF  =  (count of a salivary gland at the maximum uptake minute – count of a salivary gland at the minimum uptake minute after vitamin C) / count of background of the salivary gland at the maximum uptake minute × 100%;
(4) excretion ratio (ER)
  ER  =  (count rate of a salivary gland at maximum uptake minute – count rate of a salivary gland at the minimum uptake minute) / count rate of a salivary gland at the maximum uptake minute × 100%.

UI and UF reflected the uptake function of salivary gland, EF and ER reflected the secretion function.

### 2.4 Statistical analysis

All data were presented as line diagrams and mean ± standard deviation (SD). Statistical analysis was performed by using Statistical Package for Social Sciences (SPSS version 17.0, Chicago, IL) software. Wilcoxon Signed Rank Sum test was used to compare the preablation and postablation values within the same patients. *χ*^2^ test was applied to assess relationship between sex and outcome of first ^131^I therapy. Also *χ*^2^ test was conducted to assess association between sex and function of salivary glands. *P* values of <.05 were considered significant.

## 3 Results

**Figure 1:**
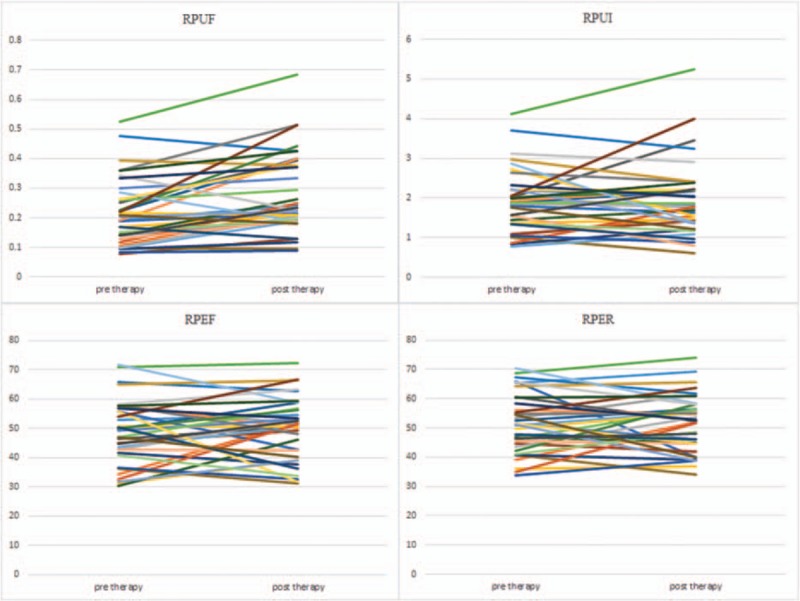
Line diagrams showing right parotid glands scintigrahy parameters. RPEF  =  right parotid ejection fraction, RPER  =  right parotid ejection ratio, RPUF  =  right parotid uptake function, RPUI  =  right parotid uptake index.

**Figure 2:**
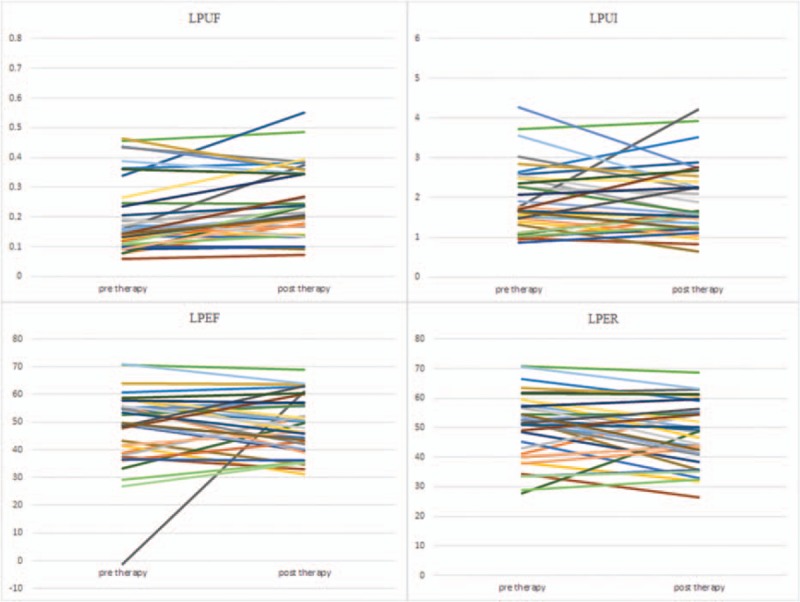
Line diagrams showing left parotid glands scintigrahy parameters. LPEF  =  left parotid ejection fraction, LPER  =  left parotid ejection ratio, LPUF  =  left parotid uptake function, LPUI  =  left parotid uptake index.

**Figure 3:**
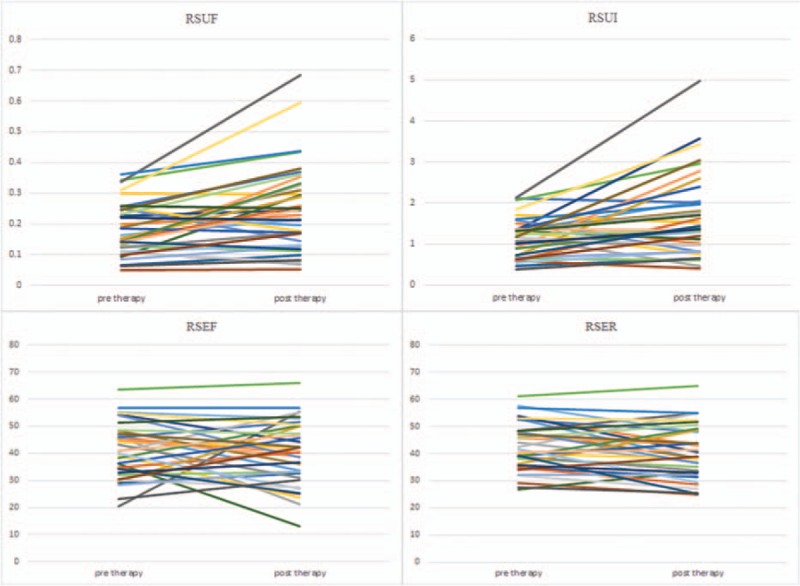
Line diagrams showing right submandibular glands scintigrahy parameters. RSEF  =  right submandibular ejection fraction, RSER  =  right submandibular ejection ratio, RSUF  =  right submandibular uptake function, RSUI  =  right submandibular uptake index.

**Figure 4:**
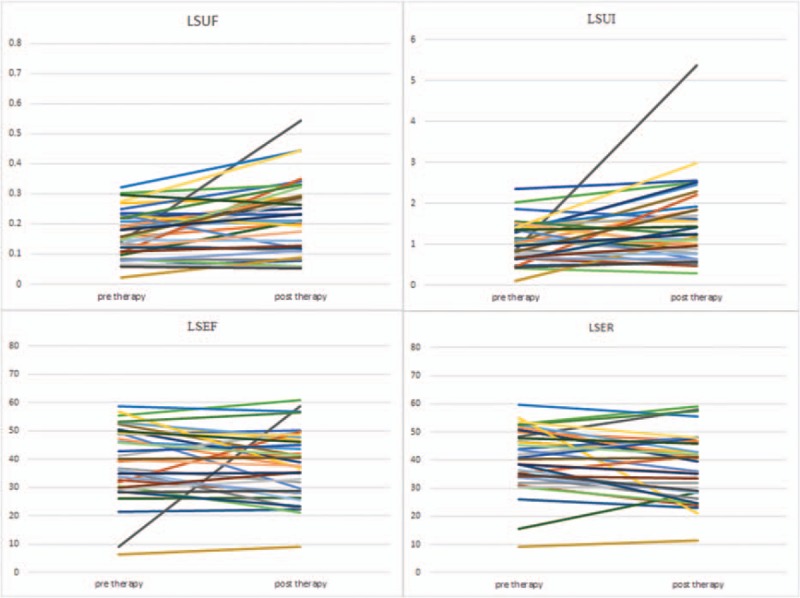
Line diagrams showing left submandibular glands scintigrahy parameters. LSEF  =  left submandibular ejection fraction, LSER  =  left submandibular ejection ratio, LSUF  =  left submandibular uptake function, LSUI  =  left submandibular uptake index.

This study was conducted on a total sample size of 36 qualified and consented DTC patients (26 females and 10 males) with age range from 23 to 69 (46.67 ± 12.58) years. All DTC patients had undergone total thyroidectomy with pathological confirmed DTC diagnosis, TNM staging T1-T3, N0-N1, M0. Line diagrams for all functional indices of salivary glands were shown in Figures 1–4.

Generally, salivary gland parameters demonstrated increased uptake functions 6 months after ^131^I therapy than before ^131^I therapy (Table 1). In specific, there was a significant increment in UF of all 4 salivary glands (all *P* < .01). Also there was a significant increment in UI of both submandibular salivary glands (*P* < .05). Therefore, our results indicated a compensatory mechanism of major salivary glands. But LS displayed decreased secretory function 6 months after ^131^I therapy (Table 1), and there was a significant decrement in ER of LS (*P* < .05).

**Table 1:**
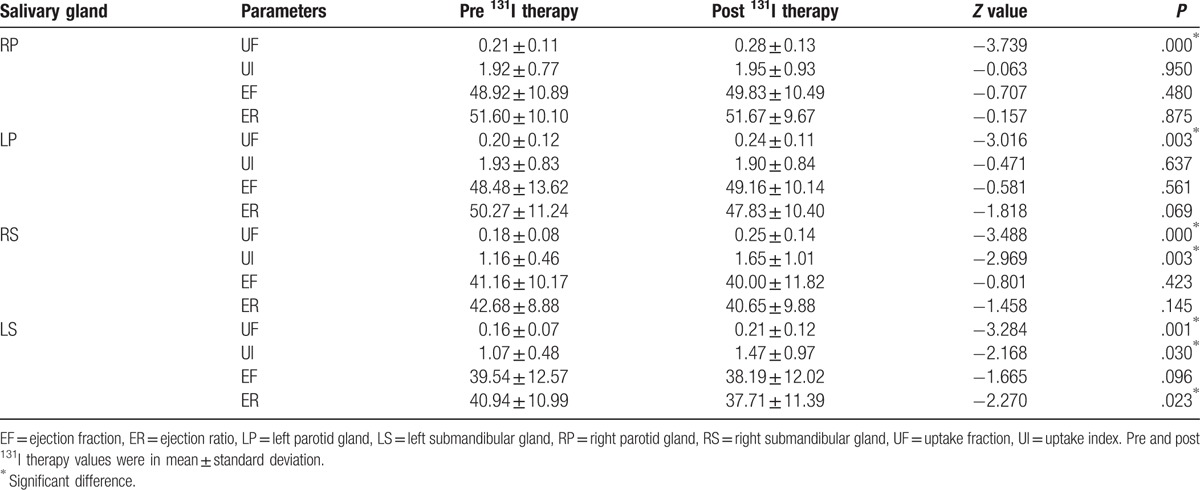
Comparisons of salivary gland parameters before and after ^131^I therapy.

Among blood parameters, Tg and TgAb exhibited a significantly decreased level 6 months after ^131^I therapy than before therapy (Table 2). There was no statistically significant association between sex and the need for a second ^131^I ablation therapy (Table 3). Also, there was no significant association between male and female on the salivary parameter changes (Table 4).

**Table 2:**
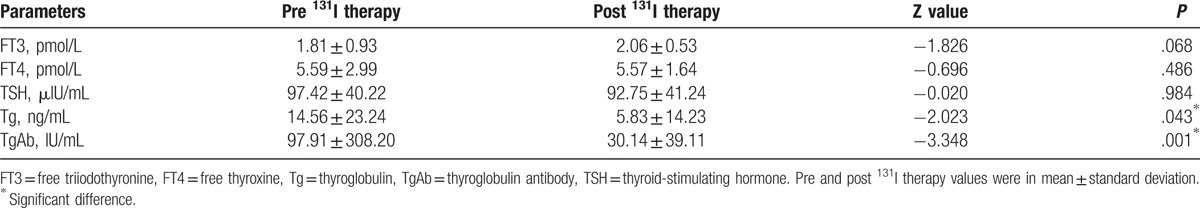
Comparisons of blood parameters pre and post ^131^I therapy.

**Table 3:**
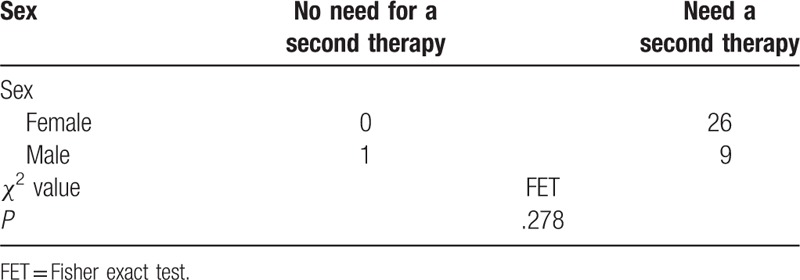
Association between sex and need for a second ^131^I therapy.

**Table 4:**
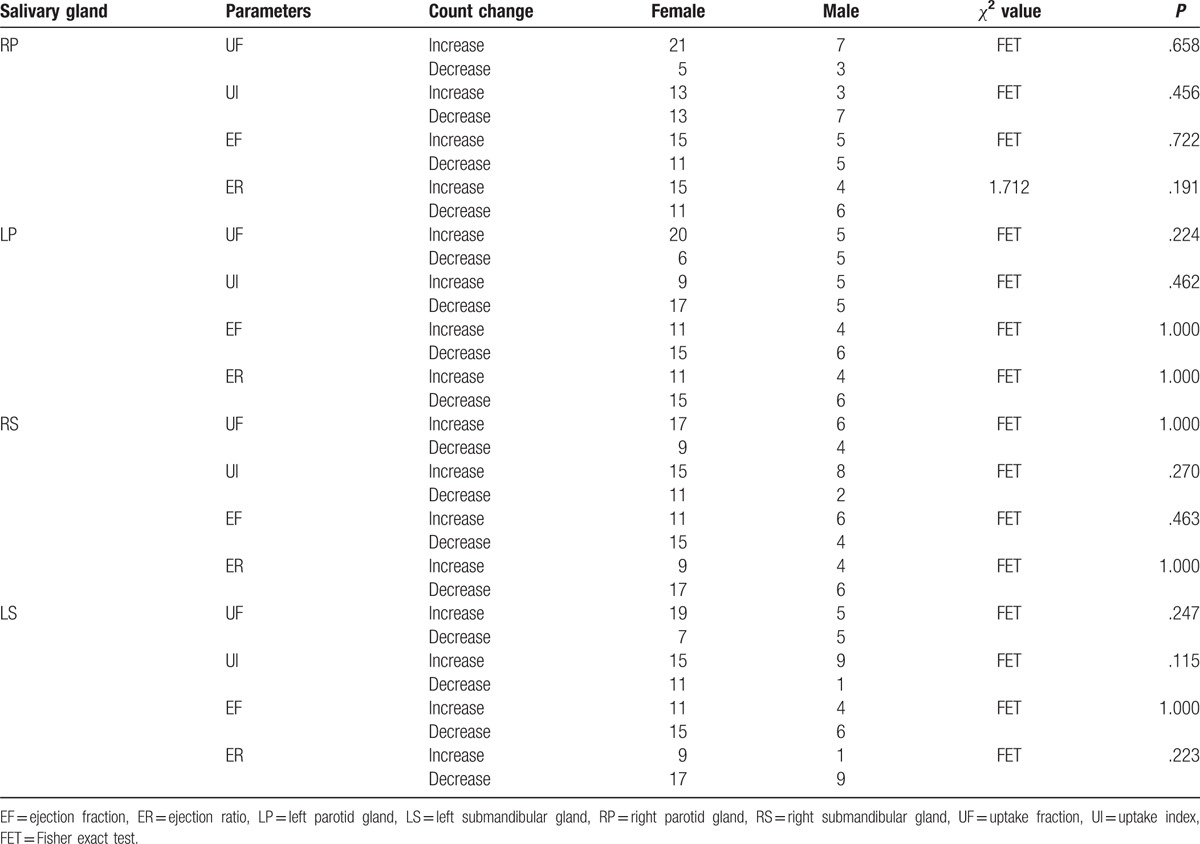
Association between gender and salivary parameter changes after ^131^I therapy.

**Figure 5:**
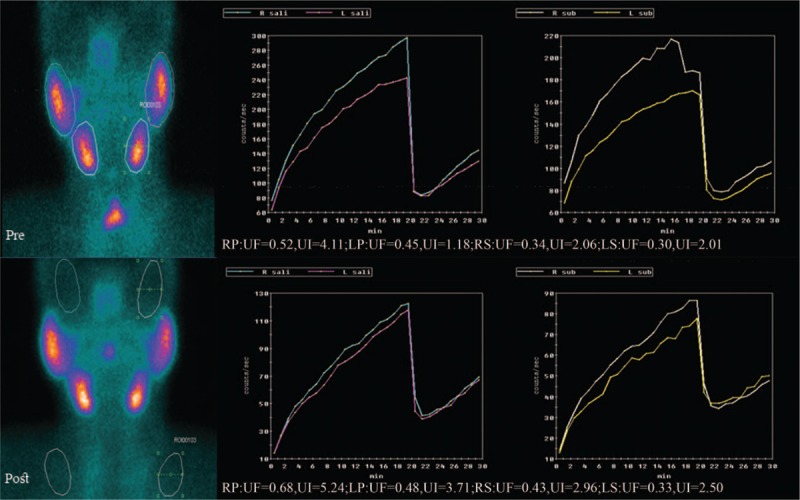
Compensated uptake functions of salivary glands after ^131^I therapy. LP  =  left parotid gland, LS  =  left submandibular gland, RP  =  right parotid gland, RS  =  right submandibular gland, UF  =  uptake function, UI  =  uptake index.

**Figure 6:**
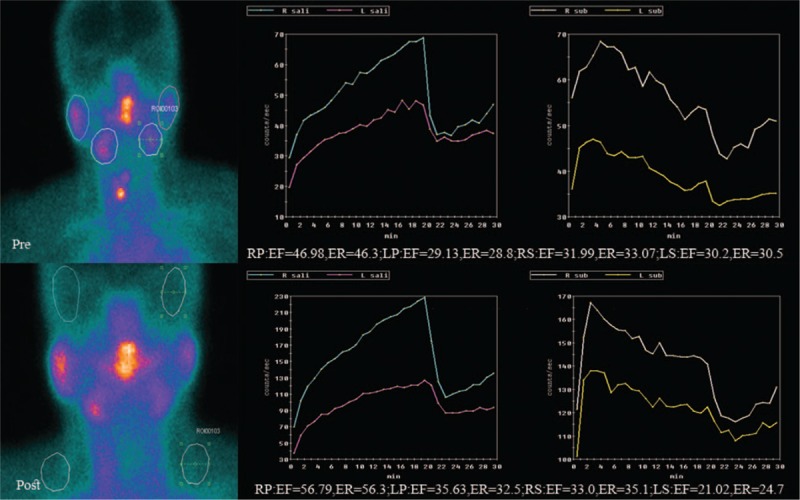
Not compensated secretory function of left submandibular gland after ^131^I therapy. EF  =  ejection fraction, ER  =  ejection ratio, LP  =  left parotid gland, LS  =  left submandibular gland, RP  =  right parotid gland, RS  =  right submandibular gland.

Two index cases, one depicted compensated uptake functions of salivary glands and the other not compensated secretory function of left submandibular gland, were shown in Figures 5 and 6, respectively.

## 4 Discussion

Salivary gland damage is a common manifestation of thyroid cancer patients after ^131^I therapy.^[27]^ Salivary gland scintigraphy was used to assess both uptake and secretory abilities of the gland after ^131^I therapy.^[10,14,28]^ In most studies, an affected gland function was found after ^131^I therapy.^[10,28]^ Yet, asymmetrical damage was reported to occur in nearly half (51%^[29]^ or 48%^[13]^) of such. ^131^I is mainly concentrated in the ductal system and the radiation may induce luminal debris that may cause ducts to narrow.^[30]^ These can lead to damage processes from obstruction of the ductal system (causing an inflammatory response in the secretory tissue, namely sialoadenitis), to glandular degeneration.^[31]^ Furthermore, salivary gland stem cells, which have been proposed to mainly reside in the excretory ducts,^[32]^ may also be affected because of the exposure to β radiation resulting in a reduced regenerative potential.^[33]^

Salivary uptake and secretory function were the focus of the current investigation. In the present study, a statistically significant difference was noted among pre- and postradiation uptake in parotid and submandibular glands. And the uptake was significantly increased in postradiation patients. Besides, there was a significant difference among pre- and postradiation secretion in left submandibular gland and the secretory function was significantly decreased in postradiation patients. There was no significant difference among pre- and postradiation secretory functions in parotid glands. The functions of bilateral submandibular glands were altered. The results of our study might be because of the initial ^131^I dose of 100 mCi. At this singular dose, the salivary glands might increase the uptake by compensatory mechanism to maintain the basic stability of the secretory function. Within the limits of the mild impairment, this kind of compensation was seen in parotid and submandibular glands.

To explain our findings, physiology of salivary glands should be discussed here. Human salivary glands are generally divided into 3 major salivary glands and many minor salivary glands. Salivary gland scintigraphy can be used to observe the major salivary glands of parotid and submandibular glands. Minor salivary glands are mostly scattered in the oral cavity, which are very difficult to be observed. There are at least 2 major steps during saliva formation.^[34]^ First, isotonic primary saliva is secreted into the luminal terminal parts of the gland parenchyma by the acini. Then, in the ductal systems, saliva is altered by electrolyte re-absorption to form a hypotonic secretion. In secretory granules within the cell, salivary proteins are continuously synthesized and stored. The parotid glands include primarily serous components. This is related to the secretion of salts and zymogen, which is the precursor of amylase. The submandibular gland consists a combination of serous and mucinous cells that secrete mucin.^[27,35]^ This mucin has a protective effect in response to radiation exposure.^[13,30,36–38]^ Although not totally resistant to radiation,^[39]^ the submandibular glands are slightly less sensitive to radiation because the submandibular glands can produce higher constant unstimulated levels of mucin secretion than can the parotid glands.^[25]^ The hypersecretion of mucin may block the duct and cause stasis of secretory system. This may lead to inflammation and ultimately damage to the gland itself. There is another explanation for this impairment. We know that ^131^I crosses through Na^+^/K^+^/Cl^−^ co-transport system. The function of this transport system is known to be affected by radiation during ^131^I therapy.^[40,41]^ This transport system is abundant mostly in ductal cells.^[42]^ This leads to ductal system constriction, acute periductal inflammation, and ultimately sclerosis causing impairment of secretory function. This phenomenon is clearly demonstrated in our study. When the acinar cells are irradiated and mildly injured, saliva secretion will be decreased. The uninjured acinar cells will proliferate and increase uptake to compensate which are required, to ensure adequate secretion by epithelial duct cells. And duct cells will also produce more mucin to compensate. Therefore, in our study, after ^131^I therapy, bilateral parotid and submandibular gland uptake was increased, whereas the secretion remained somewhat unchanged. So, we can think this was starting of compensatory mechanism to recover the cells damaged by ^131^I therapy or the hypertrophy of uninjured cells.^[43,44]^ The compensatory function of LS was manifested as increase in UF and UI of LS itself and of contralateral RS. Both actions, compensatory hypertrophy of cells and hypersecretion of mucin, lead to obstruction of ducts. This in turn leads to stasis and inflammation of LS. This leads to damage of LS. The damage of LS was manifested as decrease in ER. An et al^[25]^ also demonstrated that excretion of submandibular glands significantly reduced after ^131^I treatment.

The compensatory mechanisms of less irradiated cells of salivary glands in humans were not proved experimentally. This has been demonstrated in animal models though. Assessment of uptake functions of salivary glands in humans by sialometry or sialography techniques is difficult to use clinically because cannulization of all 4 gland ducts is very painful procedure for patients. Similarly, glandular biopsies in human are also not feasible as patients have to undergo surgery twice, one for thyroidectomy and other for biopsy itself. So we have to postulate the hypothesis of increased uptake function from research done on animal models. Elmer et al^[45]^ presented the results that an contralateral increased secretory activity is followed by glandular hypertrophy after extirpation of one of the salivary glands. Poradovskaia et al^[46]^ stated that after burn or resection of one submandibular salivary gland, the contralateral gland responded by an increase of proliferation of the contralateral gland cells accompanied by an increase in the size of the cells and the nuclei whose area enlarged by 10% and 17%, respectively. Yagil et al^[47]^ concluded that highest rate of compensatory proliferation took place in the intercalated ducts and extends, to some degree, toward both acini and granular ducts. In the study by Burlage et al,^[43]^ the observed induction of proliferation of acinar and intercalated duct cells by pilocarpine pretreatment principally could explain the observed enhanced compensatory response in salivary glands. We assumed that compensated hypertrophied cells would cause increase in uptake function. Beside the above mechanism, another probability might be because of dietary habits of Chinese people. Chinese population consumes various herbals as an integral part of their daily diet. Among them the Gingko biloba and Ginseng tea are most common, both of which have well-known radioprotective effects^[48,49]^ and could not be excluded from study groups.

The results of our study showed that with no previous dysfunctions and in the case of a certain amount of single radiation therapy, the salivary glands would be compensated to ensure the constant secretion of saliva. If some patients had inadequate compensatory capacity or the dose of ^131^I is too high, then the function of the salivary gland secretion may decrease.^[25]^ In our study, there was a decrease in secretory function of left submandibular gland as well. This may be the beginning of failure of compensatory response after ^131^I therapy. This is the indication to consider radioprotective agents to the patients during ^131^I therapy. Also, the results of our study depicted that there were no relationship between sex to salivary parameter changes and need for a second ^131^I therapy.

There were some limitations in our study. First, we had only limited number of patients, which was a major restriction of the present research. Second, the follow-up period was also relatively limited. It needs many years of follow-up to measure the actual degree of damage by subsequent ^131^I. Third, we did not compare groups with different dosages because of limited number of patients, as salivary gland dysfunction is directly proportion to the dosage of ^131^I used. Nevertheless, as we had compared all available parameters (namely UF, UI, EF, and ER) for precision of the comparisons, the external validity of these findings is anticipated to be high.

## 5 Conclusions

This study provided a quantitative comparison of salivary scintigraphy parameters after ^131^I therapy. The results showed a significant difference in uptake function of bilateral parotid glands. Furthermore, the results depicted no sex difference on therapeutic outcome and salivary gland dysfunction.
